# Supplementary material for: Genome-Wide Identification and Expression Analysis of NAC Gene Family Members in Seashore Paspalum Under Salt Stress
Source: Plants (Basel). 2024 Dec 23;13(24):3595. doi: 10.3390/plants13243595 (PMC11678376; doi:10.3390/plants13243595)

Group 1

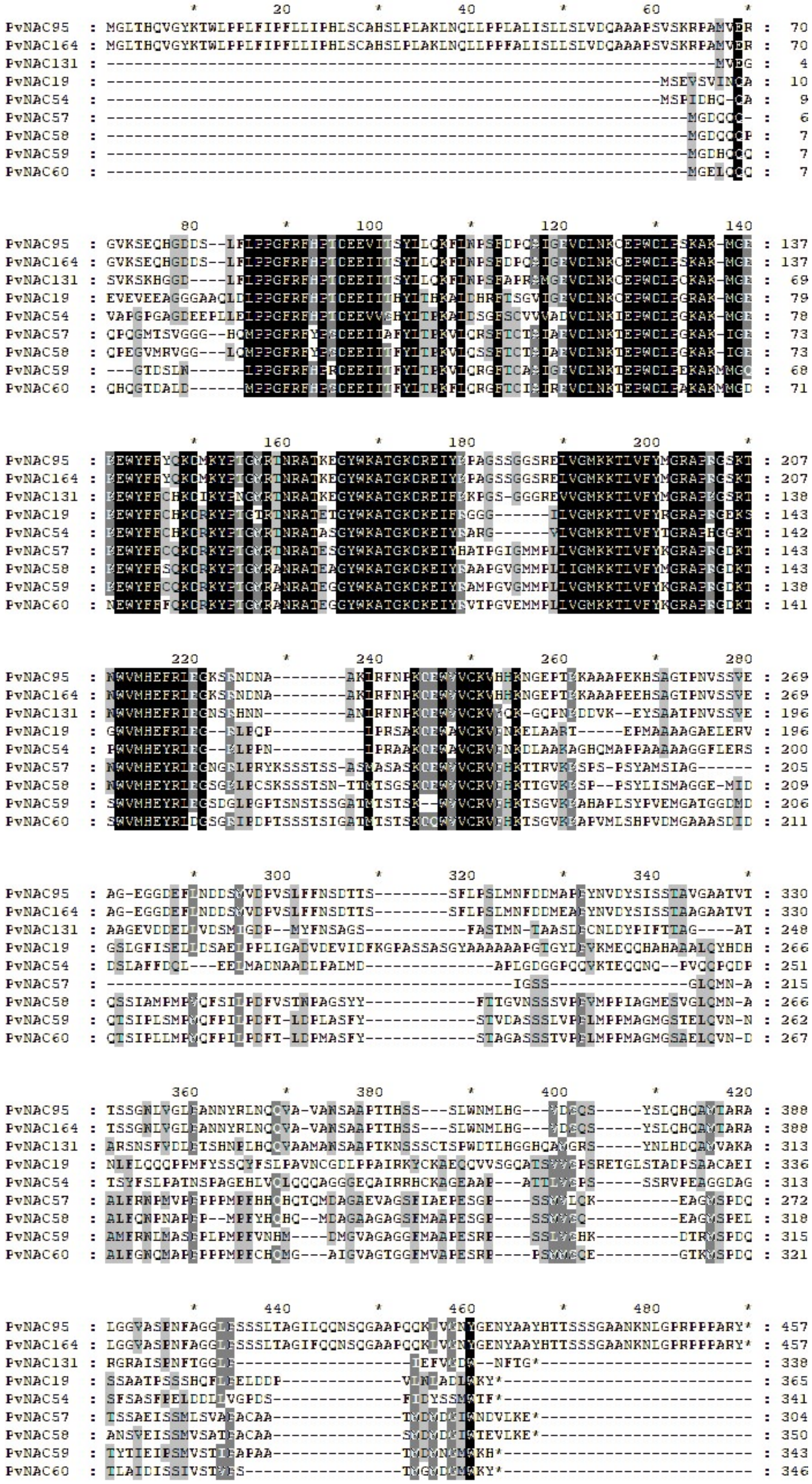
* 20 * 40 * 60 *
PvNAC95 : MGLTHQVGYKTWLPPLFIPFLLIPHLSCAHSLPLAKLNQLLPPLALISLLSLVDQAAAPSVSKRPAMVER : 70
PvNAC164 : MGLTHQVGYKTWLPPLFIPFLLIPHLSCAHSLPLAKLNQLLPPFALISLLSLVDQAAAPSVSKRPAMVER : 70
PvNAC131 : --MVEG : 4
PvNAC19 : --MSEVSVINCA : 10
PvNAC54 : --MSPIDHQ-CA : 9
PvNAC57 : --MGDQQC- : 6
PvNAC58 : --MGDQQCP : 7
PvNAC59 : --MGDHQCQ : 7
PvNAC60 : --MGELQCQ : 7
80 * 100 * 120 * 140
PvNAC95 : GVKSEQHGDDS--LPLPPGFRFHPTDEEVITSYLLQKFINPSFDPQAIGEVCLNKCEPWDLESKAK-MGE : 137
PvNAC164 : GVKSEQHGDDS--LPLPPGFRFHPTDEEIITSYLLQKFINPSFDPQAIGEVCLNKCEPWDLESKAK-MGE : 137
PvNAC131 : SVKSKHGGD----LPLPPGFRFHPTDEEIITSYLLQKFINPSFAPRAMGEVCLNKCEPWDLPCKAK-MGE : 69
PvNAC19 : EVEVEEAGGGAAQLDLPPGFRFHPTDEEIITHYLTHKALDHRFTSGVIGEVCLNKCEPWDLPGKAK-MGE : 79
PvNAC54 : VAPGPGAGDEEPLLELPPGFRFHPTDEEVVSHYLTPKALDSGFSCVVVADVCLNKIEPWDLPGKAK-MGE : 78
PvNAC57 : CPQGMTSVGGG--HQMPPGFRFYPSDEEIIAFYLTPKVLCRSFTCTAIAEVCLNKTEPWDLPGKAK-IGE : 73
PvNAC58 : CPEGVMRVGG---IQMPPGFRFYPSDEEIITFYLTPKVLCSSFTCTAIAEVCLNKTEPWDLPGKAK-IGE : 73
PvNAC59 : ---GTDSIN------LPPGFRFHPRDEEIITFYLTPKVLCRGFTCAAIGEVCLNKTEPWDLPEKAKMMGQ : 68
PvNAC60 : CHQGTDALD------MPPGFRFHPSDEEIITFYLTPKFLCRGFTCIAIREVCLNKTEPWDLPAKAKMMGD : 71
* 160 * 180 * 200 *
PvNAC95 : KEWYFFYQKDMKYPTGYRTNRATKEGYWKATGKDREIYKPAGSSGGSREIVGMKKTIVFYMGRAPRGSKT : 207
PvNAC164 : KEWYFFYQKDMKYPTGYRTNRATKEGYWKATGKDREIYKPAGSSGGSREIVGMKKTIVFYMGRAPRGSKT : 207
PvNAC131 : KEWYFFCHKDIKYPNGYRTNRATKEGYWKATGKDREIFKKPGS-GGGREVVGMKKTIVFYMGRAPKGSRT : 138
PvNAC19 : KEWYFFCHKDRKYPTGTRTNRATETGYWKATGKDKEIFRGGG------ILVGMKKTIVFYRGRAPRGEKS : 143
PvNAC54 : KEWYFFCHKDRKYPTGYRTNRATASGYWKATGKDKEIYRARG------VLVGMKKTIVFYTGRAPHGGKT : 142
PvNAC57 : KEWYFFCQKDRKYPTGYRTNRATEAGYWKATGKDKEIYHATPGIGMMPLIVGMKKTIVFYKGRAPRGDKT : 143
PvNAC58 : KEWYFFSQKDRKYPTGYRANRATEAGYWKATGKDKEIYRAAPGVGMMPLIIGMKKTIVFYMGRAPRGDKT : 143
PvNAC59 : KEWYFFCQKDRKYPTGYRANRATEGGYWKATGKDKEIYRAMPGVGMMPLIVGMKKTIVFYKGRAPRGDKT : 138
PvNAC60 : NEWYFFCQKDRKYPTGYRANRATEGGYWKATGKDKEIYRSVTPGVEMMPLIVGMKKTIVFYKGRAPRGDKT : 141
220 * 240 * 260 * 280
PvNAC95 : NWVMHEFRIEGKSSNDNA---------AKIRFNPKQEWVVCKVHHKNGEPTSKAAAPEKHSAGTPNVSSVE : 269
PvNAC164 : NWVMHEFRIEGKSSNDNA---------AKIRFNPKQEWVVCKVHHKNGEPTSKAAAPEEHSAGTPNVSSVE : 269
PvNAC131 : NWVMHEFRIEGNSSHNN----------ANIRFNPKQEWVVCKVYQK-GQPNSDDVK--EYSAATPNVSSVE : 196
PvNAC19 : GWVMHEFRIEG--RLPCP----------IPRSAKQEWAVCKVFNKELAARTT-----EPMAAAAGAELERV : 196
PvNAC54 : PWVMHEYRIEG--KLPPN----------IPRAAKQEWAVCRVFNKDLAAKAGHQMAPPAAAAAGGFLERS : 200
PvNAC57 : NWVMHEYRIEGNGKLPRYKSSSTSS-ASMASASKQEWVVCRVFHKTTRVKKSPS-PSYAMSIAG------ : 205
PvNAC58 : NWVMHEYRIEGSGKLPCSKSSSTSN-TTKTSGSKQEWVVCRVFHKTTGVKKSP--PSYLISMAGGE-MID : 209
PvNAC59 : SWVMHEYRIEGSDGLPGPTSNSTSSGATKTSTSK--WVVCRVFHKTSGVKKAHAPLSYPVEMGATGGDMD : 206
PvNAC60 : SWVMHEYRIDGSGKIPDPTSSSTSIGATKTSTSKQQWVVCRVFHKTSGVKKAPVMLSHPVDMGAAASDID : 211
* 300 * 320 * 340 *
PvNAC95 : AG-EGGDEFINDDSWVDPVSLFFNSDTTS--------SFLPSLMNFDDMAPFYNVDYSISSTAVGAATVT : 330
PvNAC164 : AG-EGGDEFINDDSWVDPVSLFFNSDTTS--------SFLPSLMNFDDMEAFYNVDYSISSTAAGAATVT : 330
PvNAC131 : AAGEVDDELIVDSMIGDP--MYFNSAGS-----------FASTMN-TAASLFCNLDYPIFTTAG----AT : 248
PvNAC19 : GSLGFISELIDSAEIPPLIGADVDEVIDFKGPASSASGYAAAAAAPGTGYLEVKMEQQHAHAAALQYHDH : 266
PvNAC54 : DSLAFFDQL---EEIMADNAADLPALMD---------------APLGDGGPQQVKTEQQNQ--PVQQPQDP : 251
PvNAC57 : ---IGSS---------------GLQMN-A : 215
PvNAC58 : QSSIAMPMPYQFSIIPDFVSTNPAGSYY------------FTTGVNSSSVPPVMPPIAGMESVGLQMN-A : 266
PvNAC59 : QTSIPLSMPVQFPIIPDFT-LDPLASFY------------STVDASSSLVPPLMPPMAGMGSTELQVN-N : 262
PvNAC60 : QTSIPLLMPYQFPIIPDFT-LDPMASFY------------STAGASSSTVPPLMPPMAGMGSAELQVN-D : 267
360 * 380 * 400 * 420
PvNAC95 : TSSGNLVGLDANNYRLNQQVA-VANSAAPTTHSS---SLWNMLHG---YDGQS-----YSLQHQAVTARA : 388
PvNAC164 : TSSGNLVGLDANNYRLNQQVA-VANSAAPTTHSS---SLWNMLHG---YDGQS-----YSLQHQAVTARA : 388
PvNAC131 : ARSNSFVDLETSHNPLHQQVAAMANSAAPTKNSSSCTSPWDTLHGGHQAYGRS-----YNLHDQAVVAKA : 313
PvNAC19 : NLPLQQQPPMFYSSQYFSLPAVNCGDLPPAIRKYCKAEQCVVSGQATSVVGPSRETGLSTADPSAACAEI : 336
PvNAC54 : TSYFSLPATNSPAGEHLVQLQQQAGGGEQAIRRHCKAGEAAP---ATIMYGPS-----SSRVPEAGGDAG : 313
PvNAC57 : ALFRNPMVPPPPPMPFHHQHQTQMDAGAEVAGSFIAEPESGP-----SSVSLQK---------EAGVSPDQ : 272
PvNAC58 : ALFQNPNAPFP--MPFYHQHQ--MDAGAGAGSFMAAPESGP----SSVSEQ----------EAGVSPEL : 318
PvNAC59 : AMFRNIMASPPLPMPFVNHM----DMGVAGAGGFMAAPESRP----SSIYEHK---------DTRVSPDQ : 315
PvNAC60 : ALFGNQMAPPPPPMPFCHQMG---AIGVAGTGGFMVAPESRP----PSVVEQE---------GTKVSPDQ : 321
* 440 * 460 * 480 *
PvNAC95 : LGGVASPNFAGGLESSSLTAGILQQNSQGAAPQQKIVVNYGENYAAYHTTSSSGAANKNLGPRPPPARY* : 457
PvNAC164 : LGGVASPNFAGGLESSSLTAGIFQQNSQGAAPQQKIVVNYGENYAAYHTTSSSGAANKNLGPRPPPARY* : 457
PvNAC131 : RGRAISPNFTGGLP-------------------IEFVLDQ--NFTG*----------------------- : 338
PvNAC19 : SSAATPSSSHQFLPELDDP--------------VLNIADLAKY*-------------------------- : 365
PvNAC54 : SFSASFPPELDDLLVGPDS---------------PIDYSSMATF*------------------------- : 341
PvNAC57 : TSSAEISSMLSVAPACAA---------------TVDVDLDINDVLKE*---------------------- : 304
PvNAC58 : ANSVEISSMVSATPACAA---------------SVDVDLDITEVLKE*---------------------- : 350
PvNAC59 : TYTIEIPSMVSTIEAPAA---------------TVDVNLDMKH*-------------------------- : 343
PvNAC60 : TLAIDISSIVSTPES-----------------TVGVDLDMKY*--------------------------- : 346

Group 2

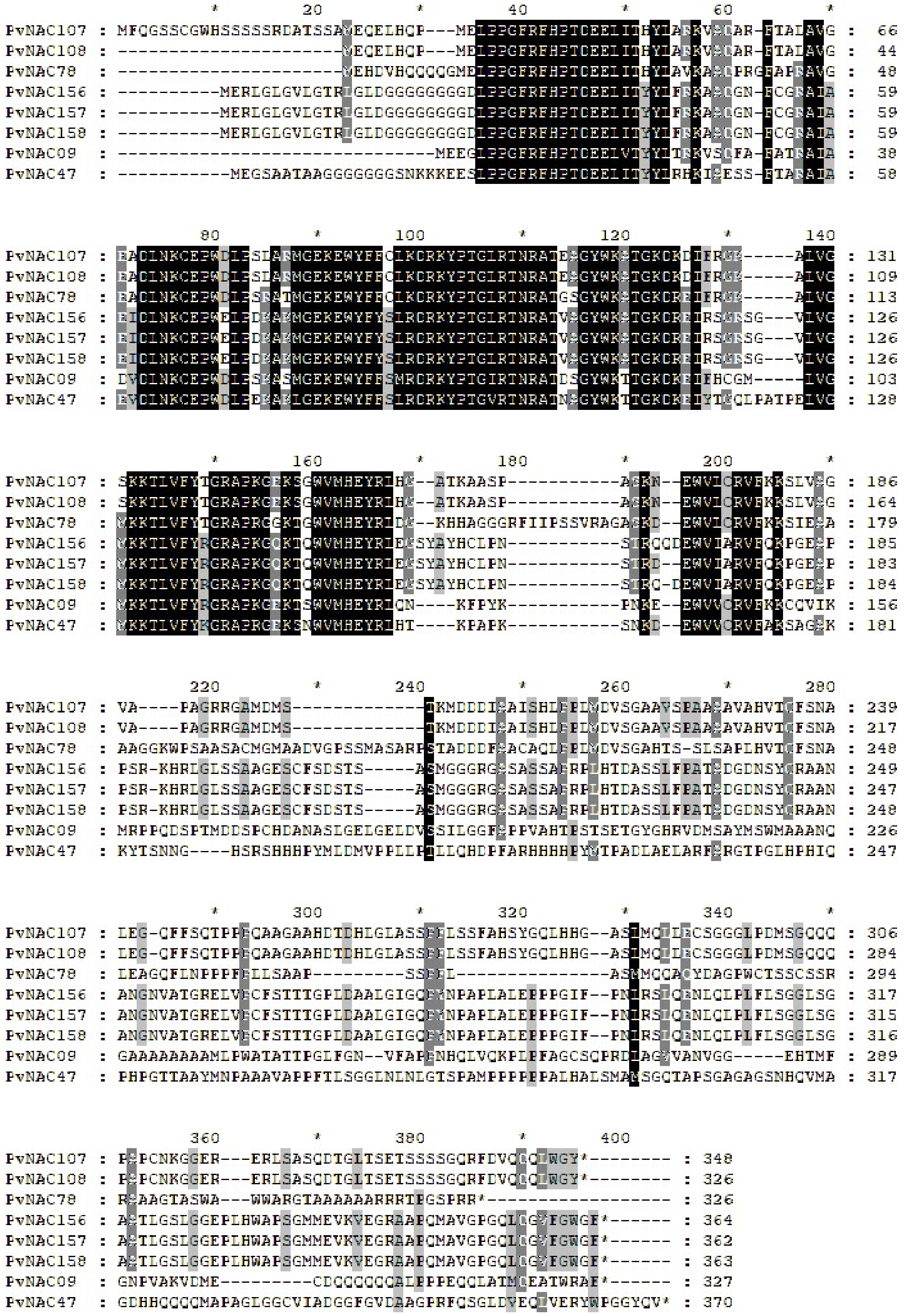

Group 3

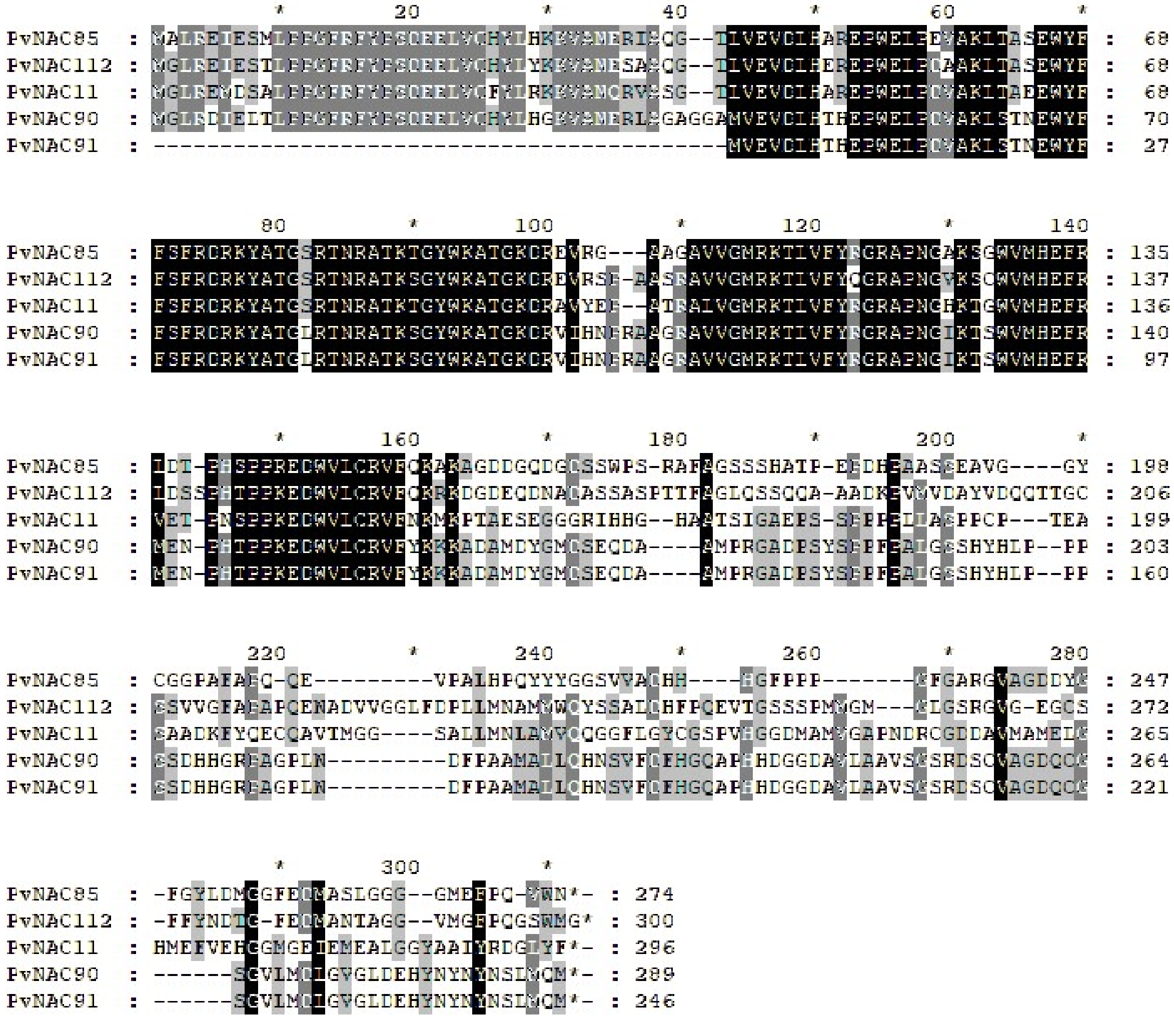

Group 4

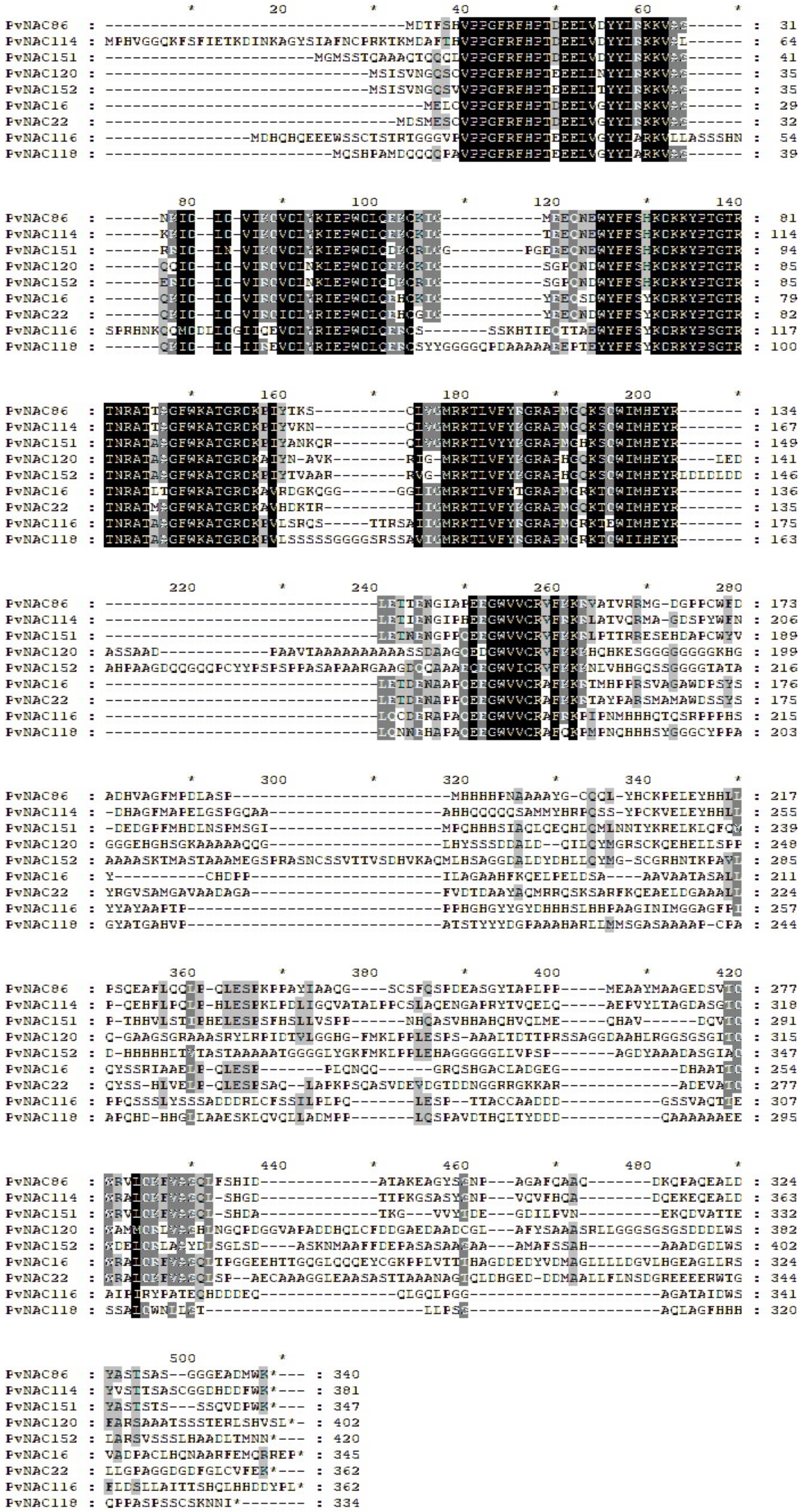
PvNAC86
PvNAC114
PvNAC151
PvNAC120
PvNAC152
PvNAC16
PvNAC22
PvNAC116
PvNAC118

Group 5

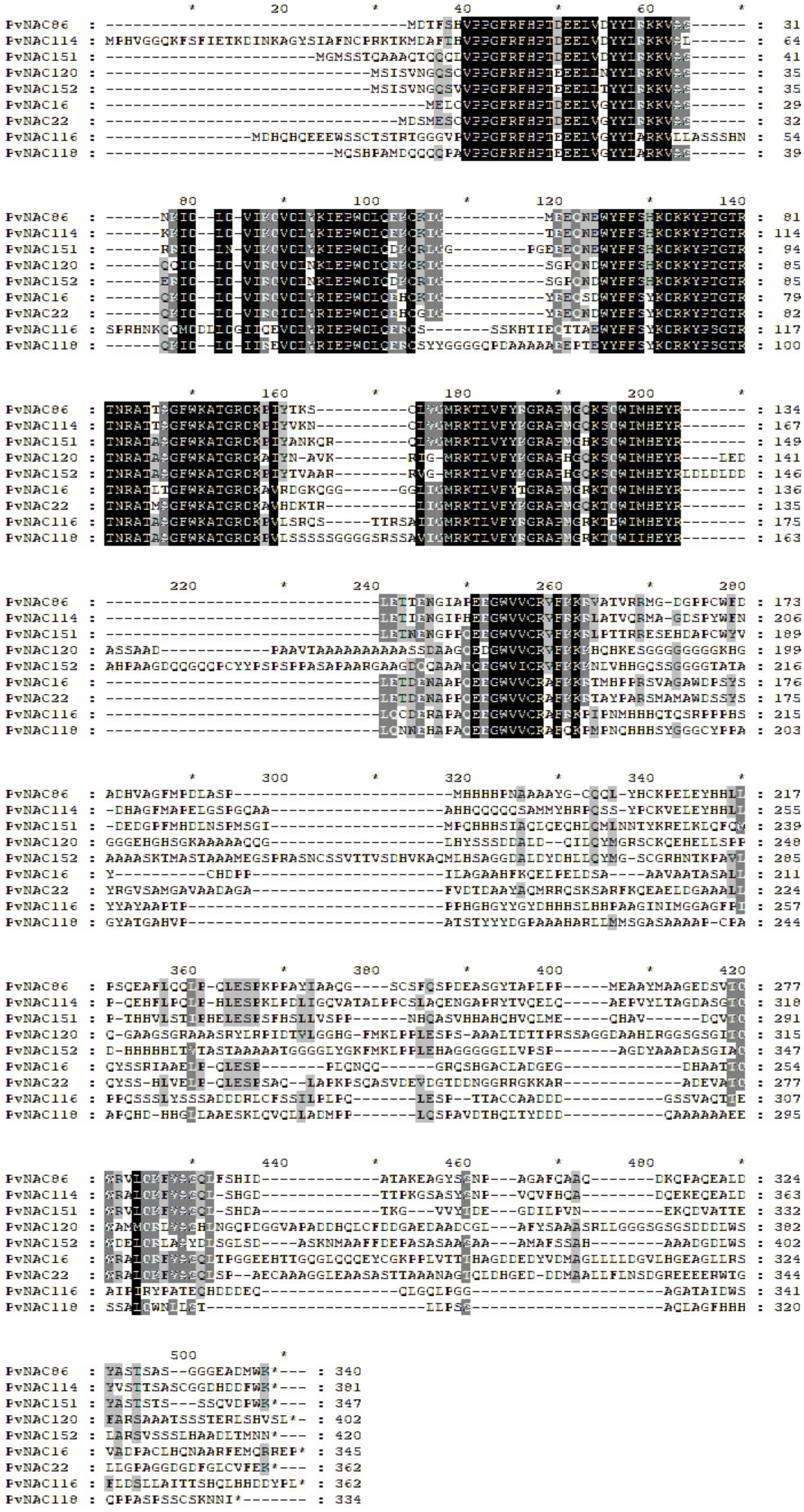
PvNAC86
PvNAC114
PvNAC151
PvNAC120
PvNAC152
PvNAC16
PvNAC22
PvNAC116
PvNAC118

Group 6

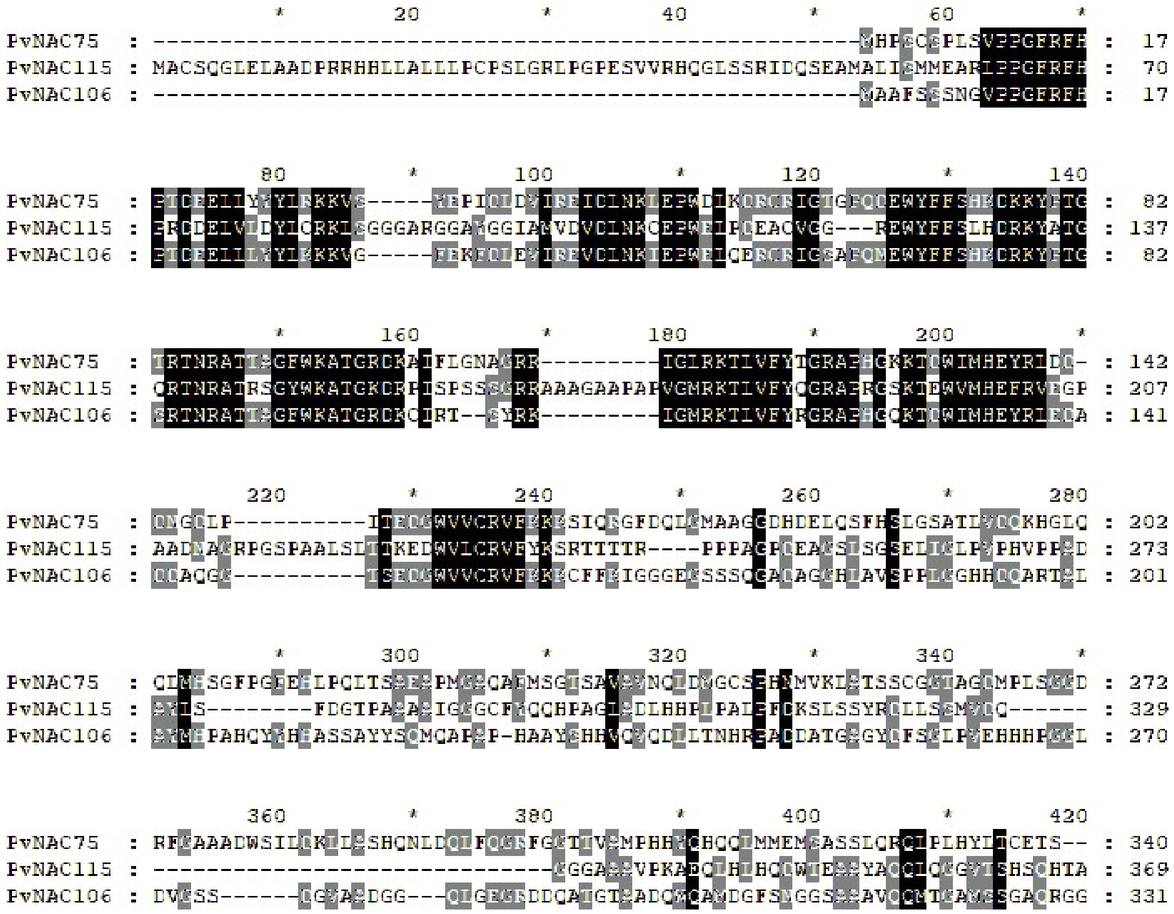

Group 7

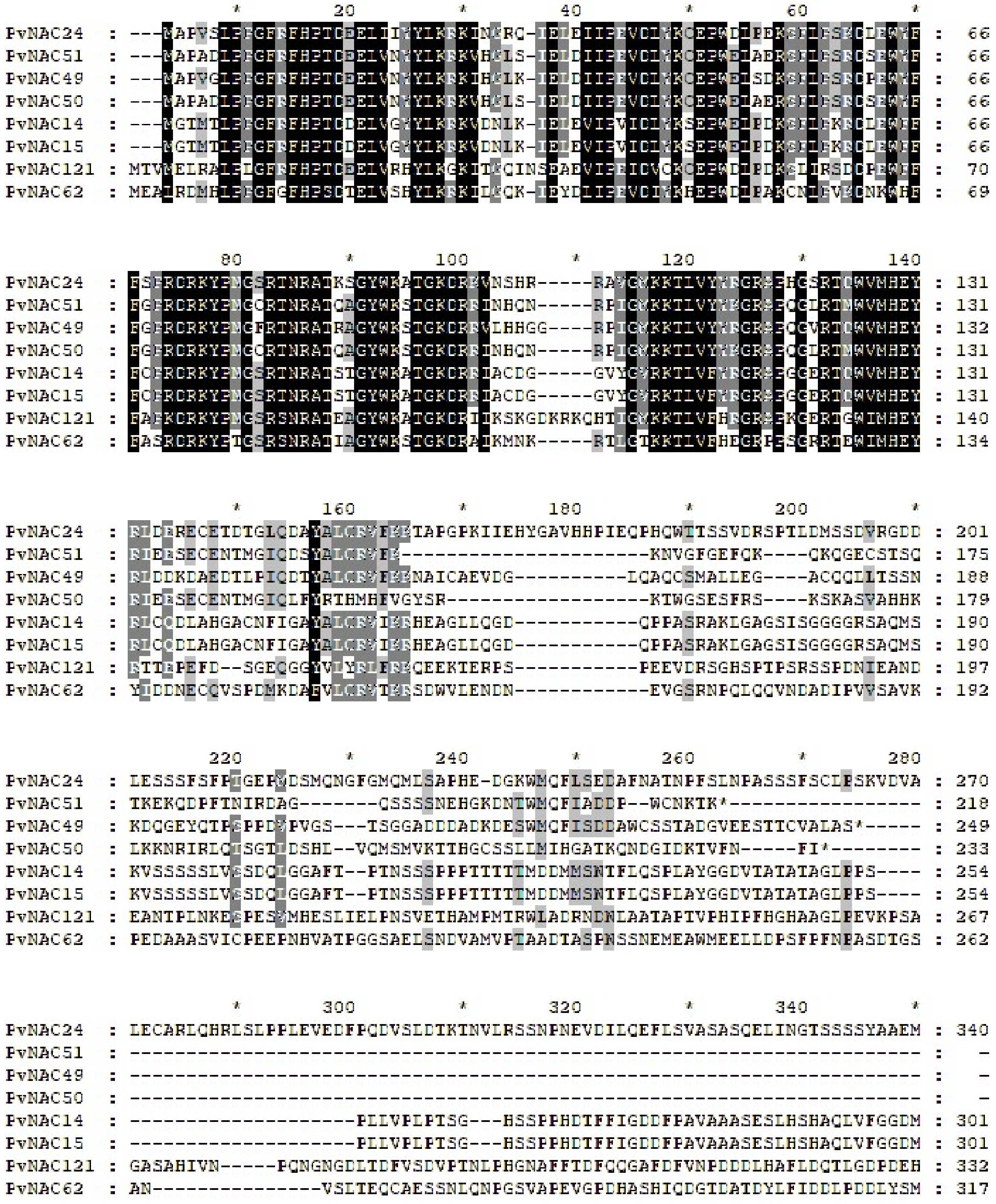

Group 8

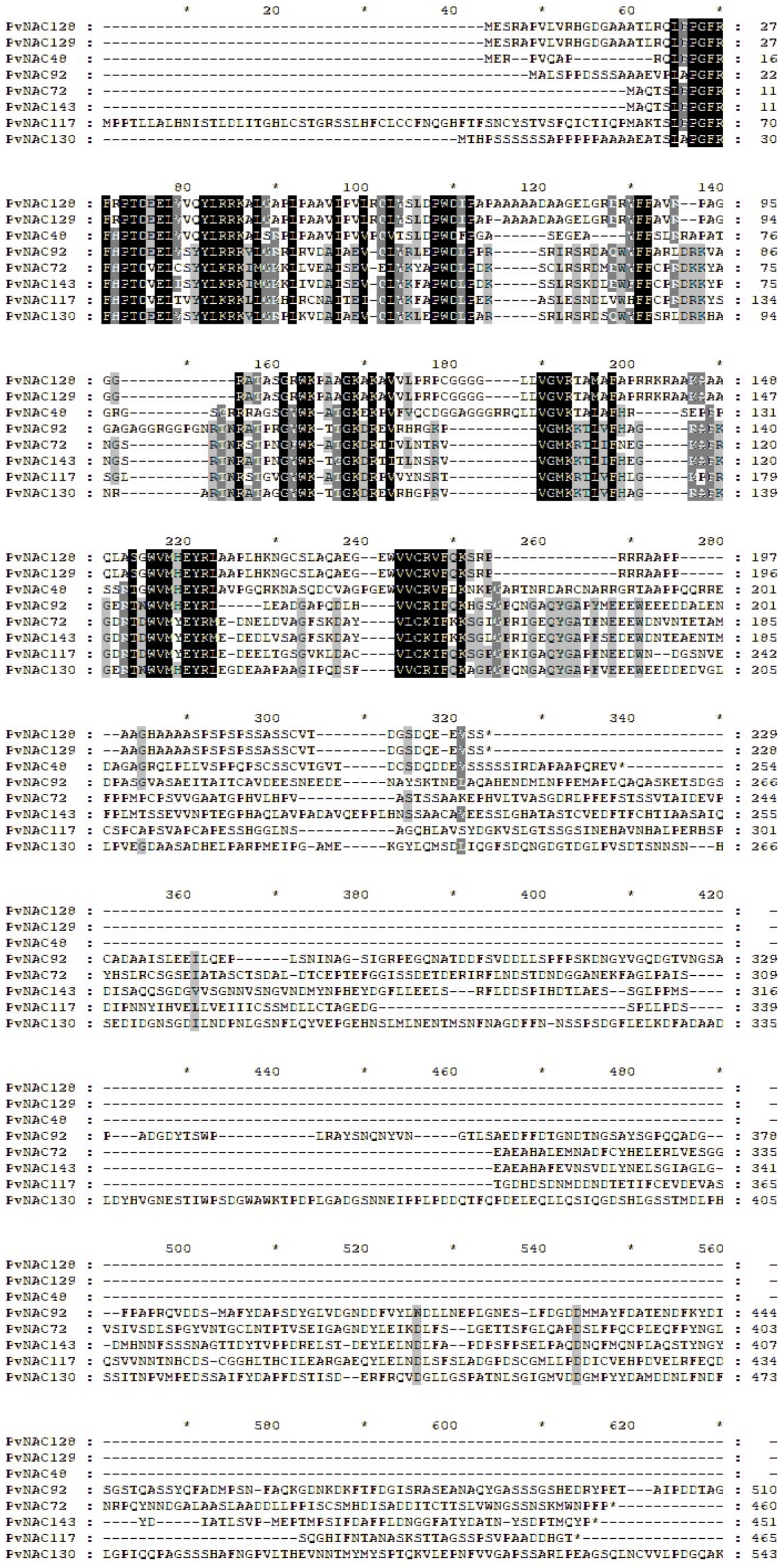

Group 9

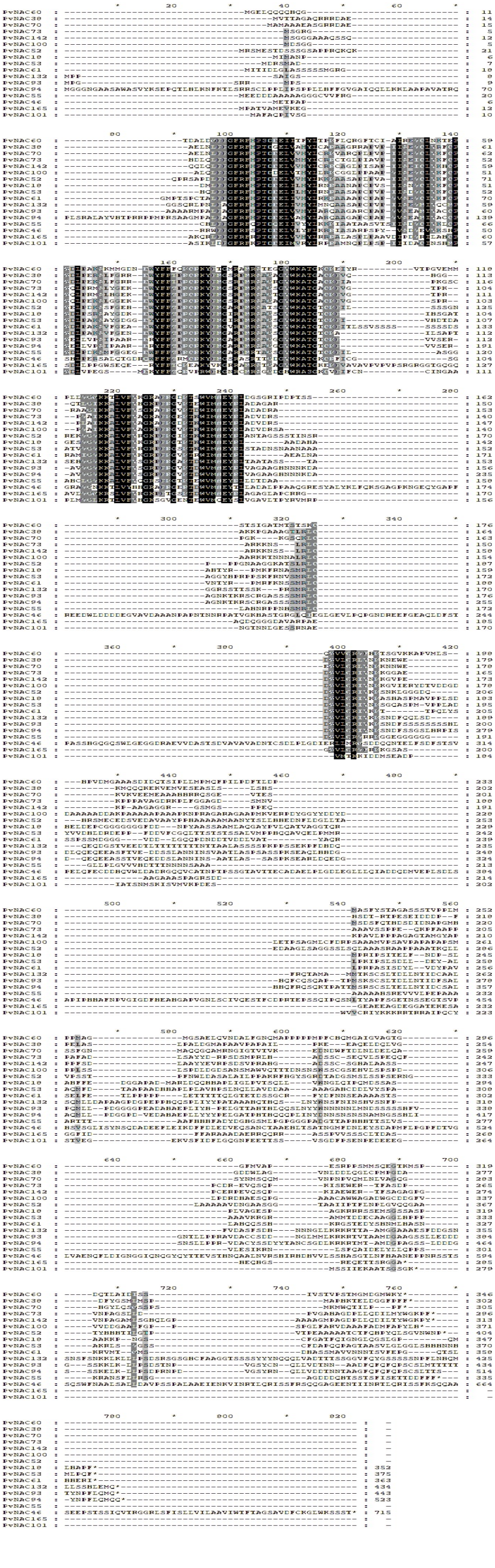

Group 10

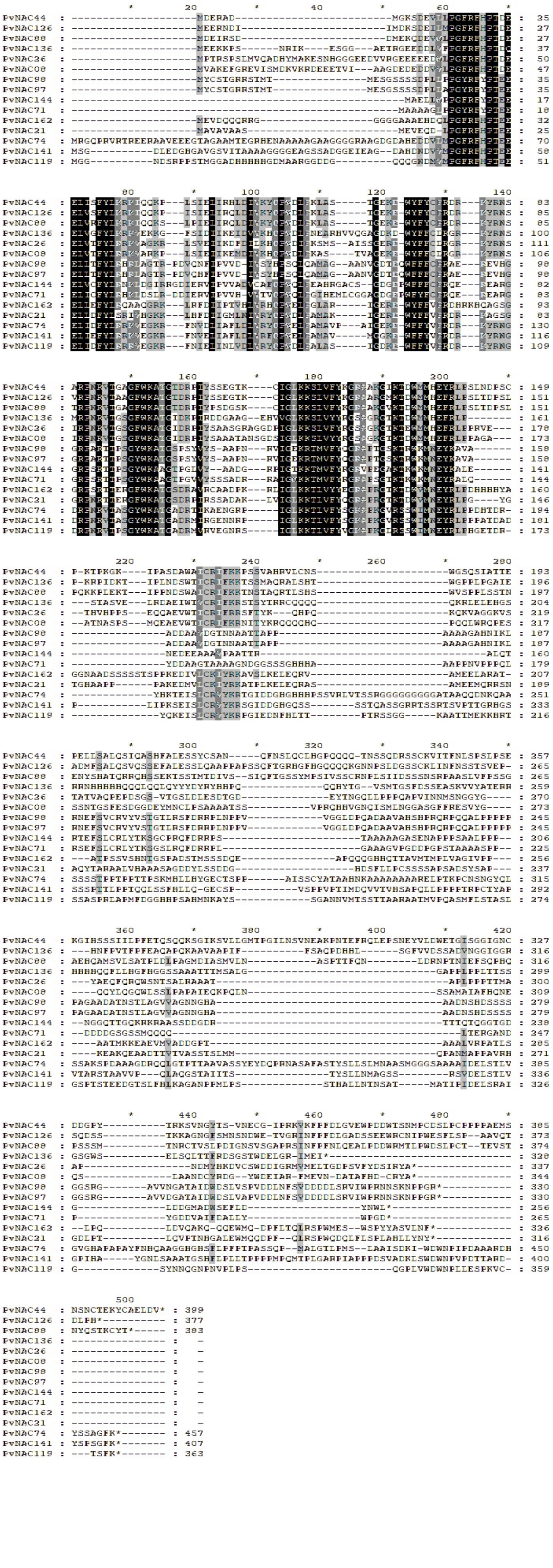

Group 11

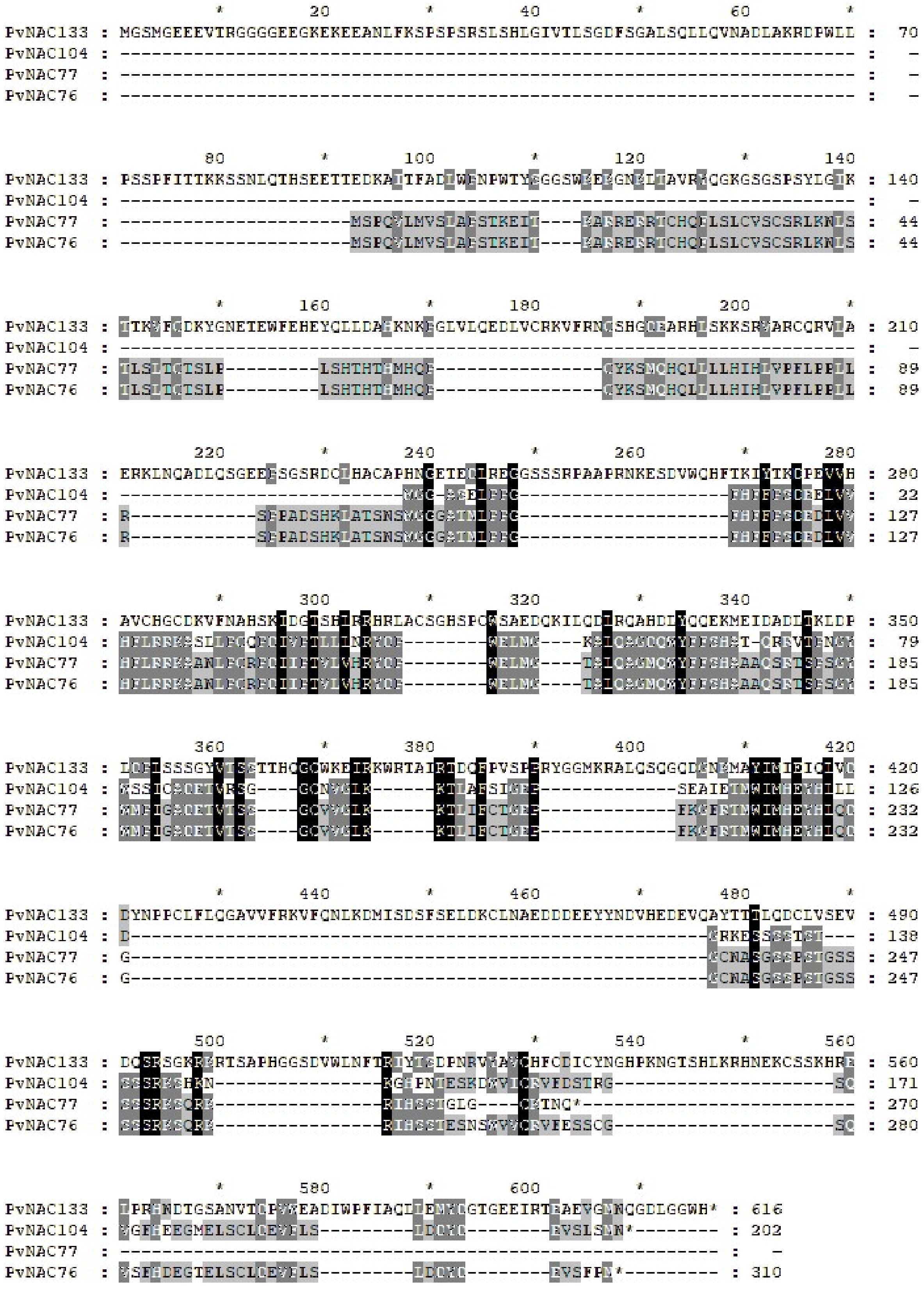

Group 12

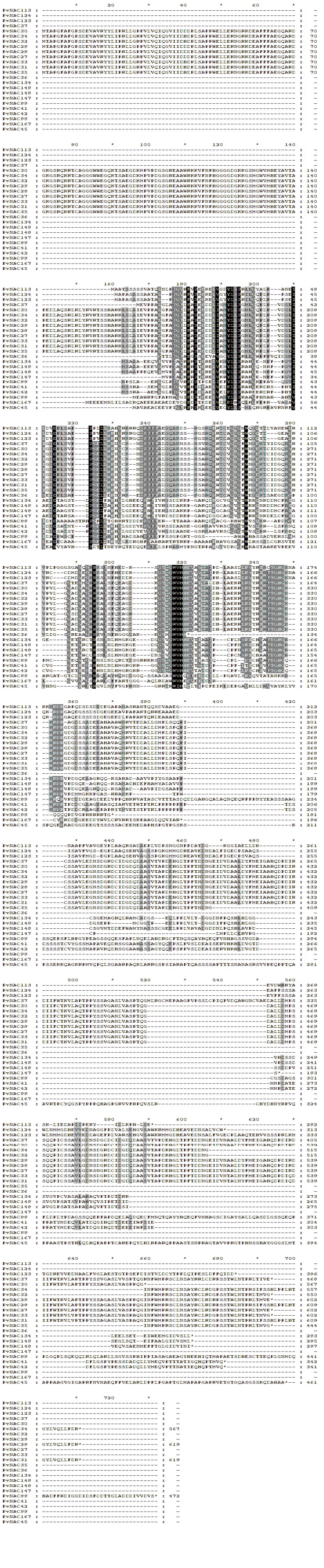

Group 13

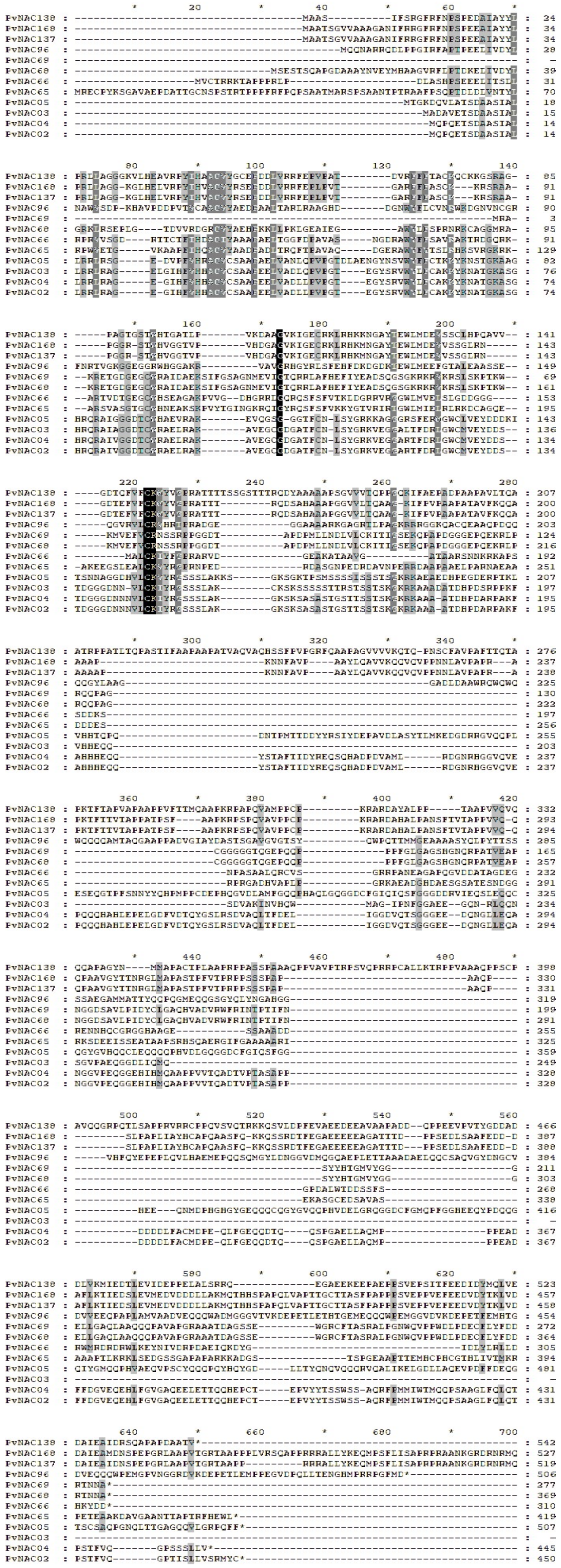

Group 14

```
                          *        20         *        40         *        60         *
PvNAC150 : -------MGMERHELVQQLRFPPGYRFVPTEEQLIDVYLRCKIEG--DPLHLD--VVNEVS--ILDW--- :  54
PvNAC140 : MEVAAGKDQDDERQLVRQLQFAPGYRFVPSEQDIVNVYLRGKIEGWKNNNLALNNVVNEVA--ILEW--- :  65
PvNAC103 : ------------------MRQQAA---ETTDAEIIEALARRRWGQPPAPSQPF--TVHDA--FIFDS--- :  42
PvNAC102 : ----MGFGPFFPPFPTFFKLRNGASPWRAVTDVQIIEALRRRRLRLPPAPSHPF--TVHDVSLYVYNLSSH :  64
PvNAC13  : ----MQMQPLVPGGFLTRHGFPPGVRFVPTELEIVSILSCRVRGNPLPSPVAT--IFHDVN--VLSF--- :  59
PvNAC12  : ---MTQQRAISVGSLITKHGFPSGVRFVPTDMEIVSILSDRVRGVPLRPRVAA--VFHDVD--ILDY--- :  60

                   80         *       100         *       120         *       140
PvNAC150 : TPDGIVETPKGYG-ENKWYFFTIRDQSS--------------SKKEKQPSRKVKAPGVTASWKATGSAVP : 109
PvNAC140 : QPGAIVEKMKAYG-TDKWYFFTIREPSS--------------SNKVKEPDRKVRVPGVTAAWKATG---- : 116
PvNAC103 : HEARIYDENGRG--AGCVYFFSEPRSTTH-----------YLERQGER------------GWKVAG---- :  83
PvNAC102 : HESKIYDEMGGGA-AGCVYLFADQ-----------------LTNKDHE------------GWKVAG---- : 100
PvNAC13  : HPKEIHEMMKQHEEERYIYFFSHRQLQK------------LLPADEKQKVPRPVRVAKNGGWKASG---- : 113
PvNAC12  : HPKEIHEMMRHDTEYRYIYFFSCRRFQTPAQGGDQRAAAVFFPEDEHQKEPRPVRVAKGGGWKPSG---- : 126

                          *       160         *       180         *       200         *
PvNAC150 : IMKTNKAATSVIVKDDGEKPGGSGDNDENNKAVVEEEIEPVLIGLKRVLTYQSTDPEQNGKWSMHEYILQ : 179
PvNAC140 : ------------------RVSAIHAKPKELGEPIDDMSKDGVIGTKRVLIYHSSDAEEHRKWSMHEYILK : 168
PvNAC103 : ----------------GTR--RLMDGGGR---EVGSKDTLVFH--------EGRW-LPRTRWAMDEFDEH : 123
PvNAC102 : ----------------GTQ--RVPDAGGS---EVGRKNTLVFH--------EGRW-LRRARWAMDEFNYT : 140
PvNAC13  : ----------------PAKLLRSPEGNGRRRFVAGRMATMVFYDTVVNEKGVKSA-SLKTNWAMNEITIP : 166
PvNAC12  : ----------------GAKPLRLPPRQG--GFVVGKMVSMVFYDEAQG-AGKGKK-QVKSNWGMHEVTIP : 176

                  220         *       240         *       260         *       280
PvNAC150 : DHDQIGQYSLQSIQRIIHSNRRKKKNGDAADIDDQEKSSSTEEPSSS---SEEPGTDQTTLAETPLKP-- : 244
PvNAC140 : GHDAIGQYALQSIQLKQHSDTKLEASAAVELQHQEATTTPTEVALKRPRRKTKRQNQKTIFTDRKKKPSM : 238
PvNAC103 : KNYQE---------REIRLYRLRRLA*------------------------------------------- : 140
PvNAC102 : KHDEESWLAC--DMVDIRLYRLRRLDDA*----------------------------------------- : 166
PvNAC13  : RDQRLSSVASNTRFYDIALYRLYSLEKKAGDTEAETSAGTGAGQALENVVSDHLPAPSMAVASCPPAG-- : 234
PvNAC12  : VKRELTDIESSTRFYNIALYRLYVLK--SGDVEAENRR-AAAGGTGKNAVSDN--CSSTAVVPCPVPR-- : 239

                          *       300         *       320         *       340         *
PvNAC150 : -TTGKTSAQDQTTSKGKGEPPMQAEGVQALQQQQQLSAAYGAPAPLLPVDAVPLQVLPSYYPGGGIAARA : 313
PvNAC140 : QAEGQMQEEEEGISPDRPVELPVTPWNQDLGALQQEQQPLVPYGALPVIPAAPLQEYPS-HPGGNHQPGA : 307
PvNAC103 : ---------------------------------------------------------------------- :   -
PvNAC102 : ---------------------------------------------------------------------- :   -
PvNAC13  : -QPFGVSTANQQLAVGPSTSAWMPPPPQHRLSIQHAQ-YYYPHDQYASFGAAQQQQLHAMPMHASIGPAA : 302
PvNAC12  : -APLRISTENQTAAVGASTSS-MPPP-QLRLSAQHQQRAHCYHRQHASFGAAQQQQQQ---LHATMAP-V : 302

                  360         *       380         *       400         *       420
PvNAC150 : SSKPMHGEGGDILSQLQQVLEQQNCNPMFLQHNIMSQFLASEYYPNNPTMAFNNQQEQPSPCSLTDGVQN : 383
PvNAC140 : SASMHQGDAHAYSFQCQDVYHQFPMQGPQMLHNRDEQPLGFTASEFFISPVTGSNVDHVG---LIQQQED : 374
PvNAC103 : ---------------------------------------------------------------------- :   -
PvNAC102 : ---------------------------------------------------------------------- :   -
PvNAC13  : HLFPLVGPPALAPVPRSPYLMPPSTLPPADVRPPVEQQQLHPATTGTTHGAG-QEVGRFG--VVTRSSYL : 369
PvNAC12  : HGGSIRQPALLFPLAP-----PPPTAP---------------------HGAG-QQFGCLGGGAFTSSPYL : 345

                          *       440         *       460         *       480         *
PvNAC150 : QPSFSGENDGEQSLGQYGVDQFLVGNTSTTTQLMNTGGWEHDGVQYQQHLNSQHYYGELSGHQIYYQQDD : 453
PvNAC140 : HDLFGVHSQHLYTEGQY-LNQNSNLFMGNTTWRSSSREHDHHQIYQHENEGAFGAQVKNTDAQYYDQSIE : 443
PvNAC103 : ---------------------------------------------------------------------- :   -
PvNAC102 : ---------------------------------------------------------------------- :   -
PvNAC13  : MPPPS-------TLPPADVRPP--VEQLPPAT-TGTHGAGKDEAGHFG-VASSPCSAPVAGSSGSPPSQQ : 428
PvNAC12  : APLPMSTTPMLVPLPANNMNPPVEVEQMAPATGDTTHGAGQEAAGHLG-VTASPPAAP-SGSSQSEQHAS : 413

                  500         *       520         *       540         *       560
PvNAC150 : GGVFGVQDQDQQRNYGCYDGQYFKDLSLQVDLFAHAGSSPDEQFIDGSEADYDSDEAFAESVKRQQDDPE : 523
PvNAC140 : QGVYGTSGQIGYGSSDQFTKGDDNSADGSFDTESFFP-----------DISYDDPHQDLVSAHRRGEQSS : 502
PvNAC103 : ---------------------------------------------------------------------- :   -
PvNAC102 : ---------------------------------------------------------------------- :   -
PvNAC13  : PATATTTTEPSPPDQDIVVPDAQENGMEVLDWLPDLA----DLDENDFRFRFTMEELMGGSVLDDDDNEP : 494
PvNAC12  : ATTEPTAEEVPPPLEEDFVPAAQDERADDADIYGGLP----DLWNP--NLVFTLEELMGEPAASDEP-AA : 476

                          *       580         *       600
PvNAC150 : DSGSDDLTSSSQSQDEQLRCSSGGLLEDVVQCHMGWHDTGI*- : 564
PvNAC140 : SLAANMSSSDHVHDSNMLPMGEPSSYTDVVSNHATCTGSWWC* : 544
PvNAC103 : ------------------------------------------- :   -
PvNAC102 : ------------------------------------------- :   -
PvNAC13  : LPMQETRDTTVVAERTSTRQESGVKN*---------------- : 520
PvNAC12  : LPMQGG--------NNRGRDENPSLY*---------------- : 494
```

Group 15

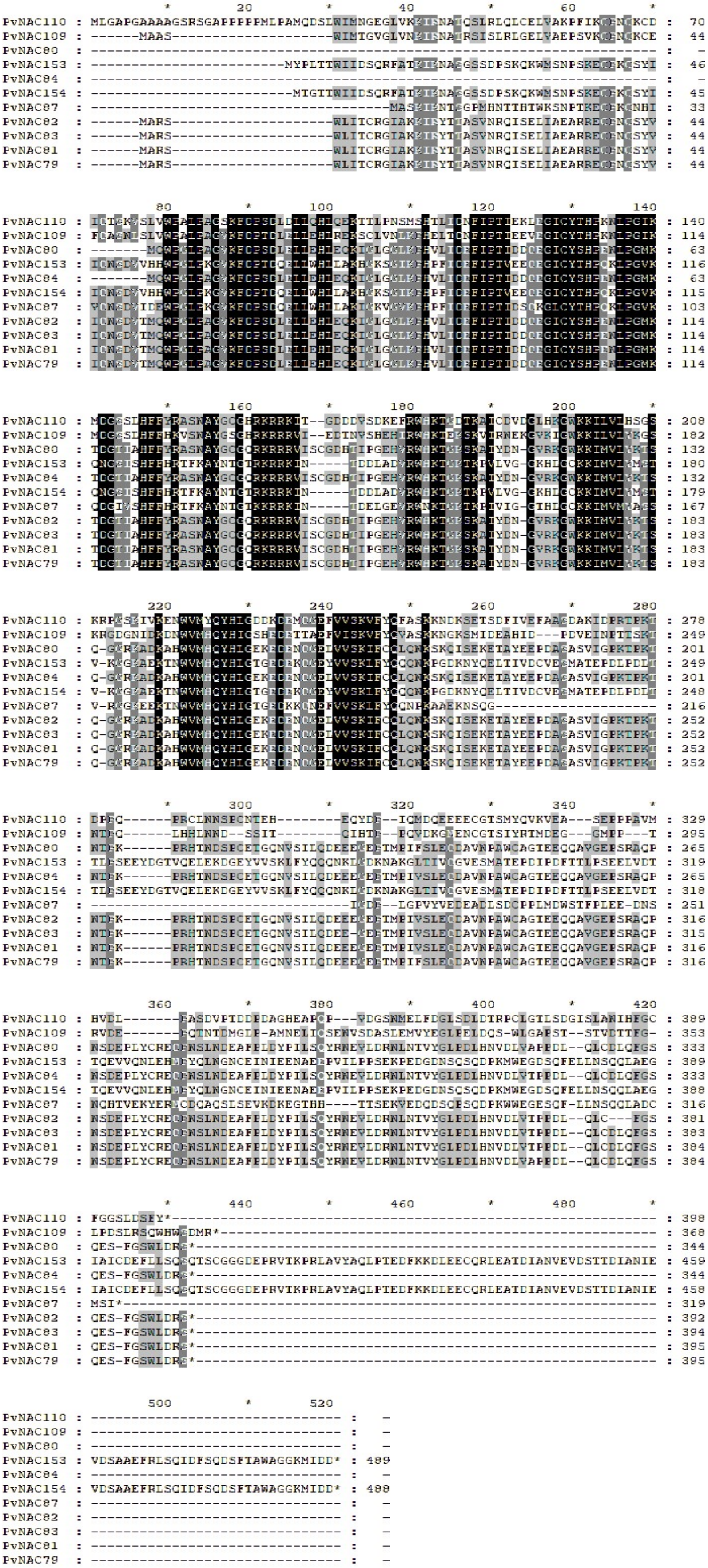

Group 16

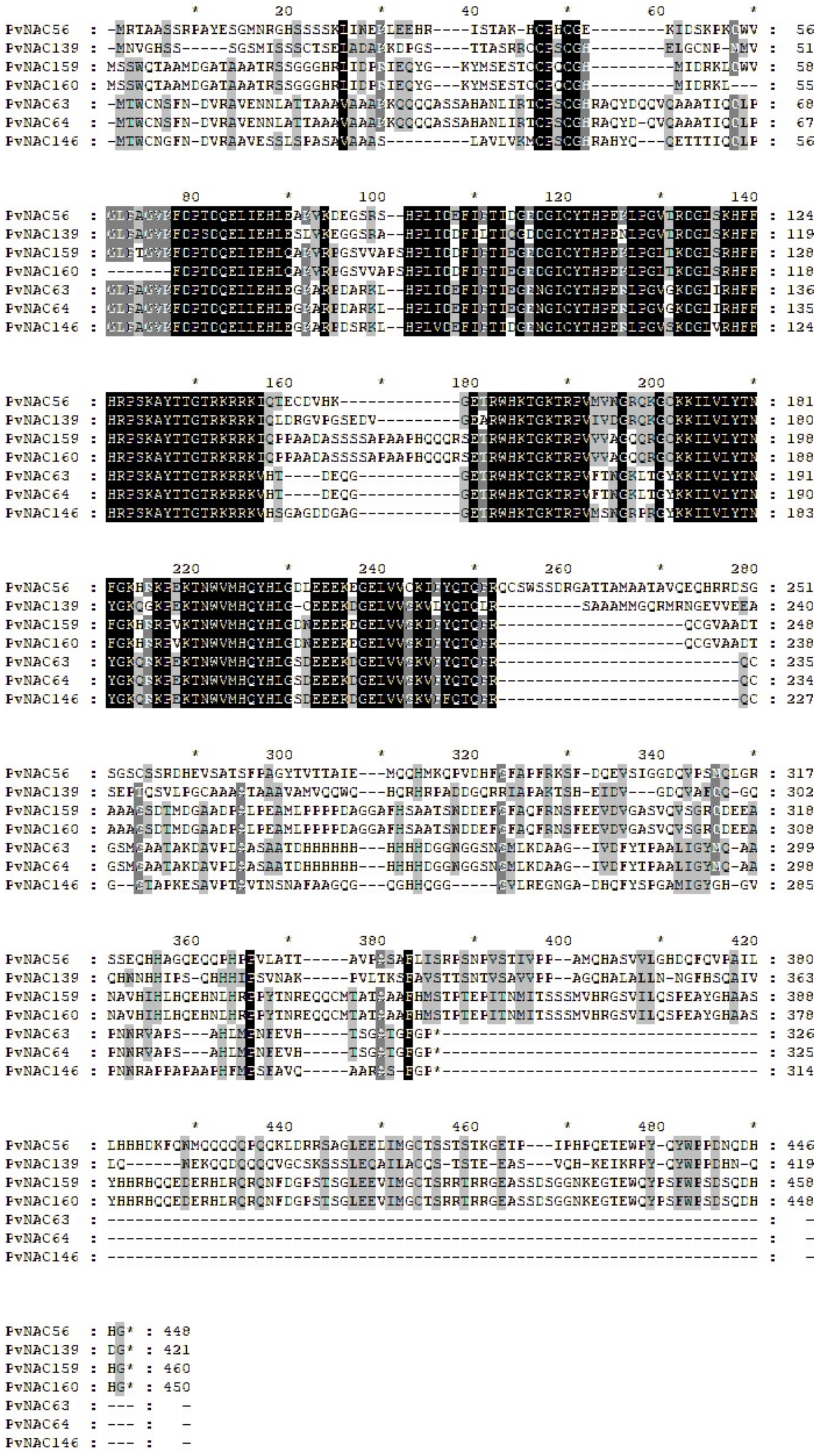
PvNAC56
PvNAC139
PvNAC159
PvNAC160
PvNAC63
PvNAC64
PvNAC146

Group 17

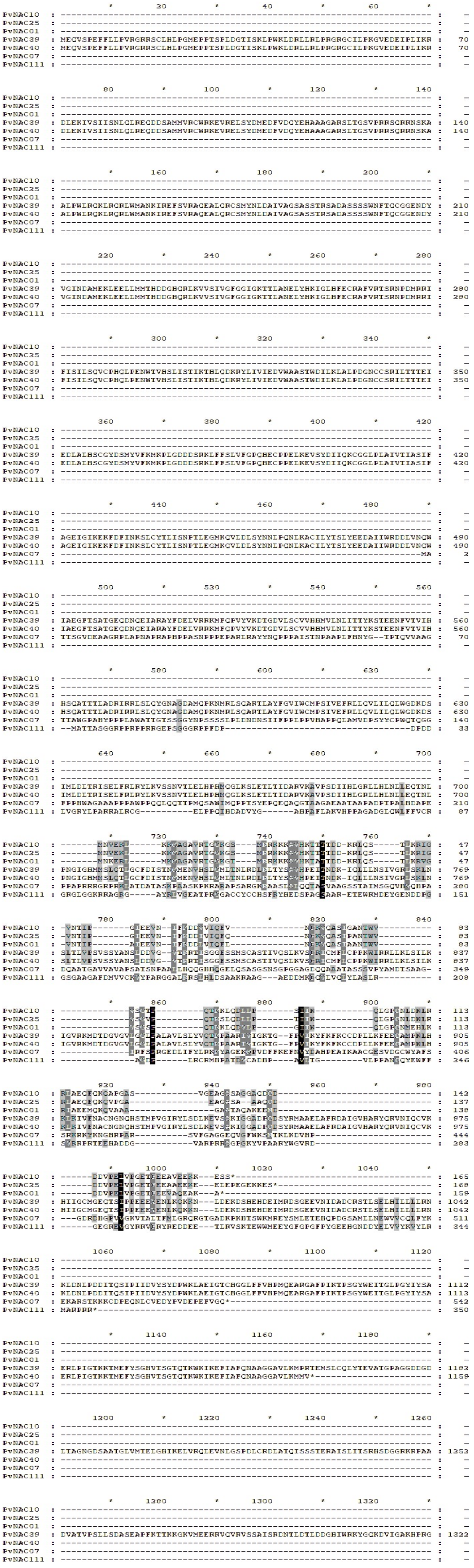

Group 18

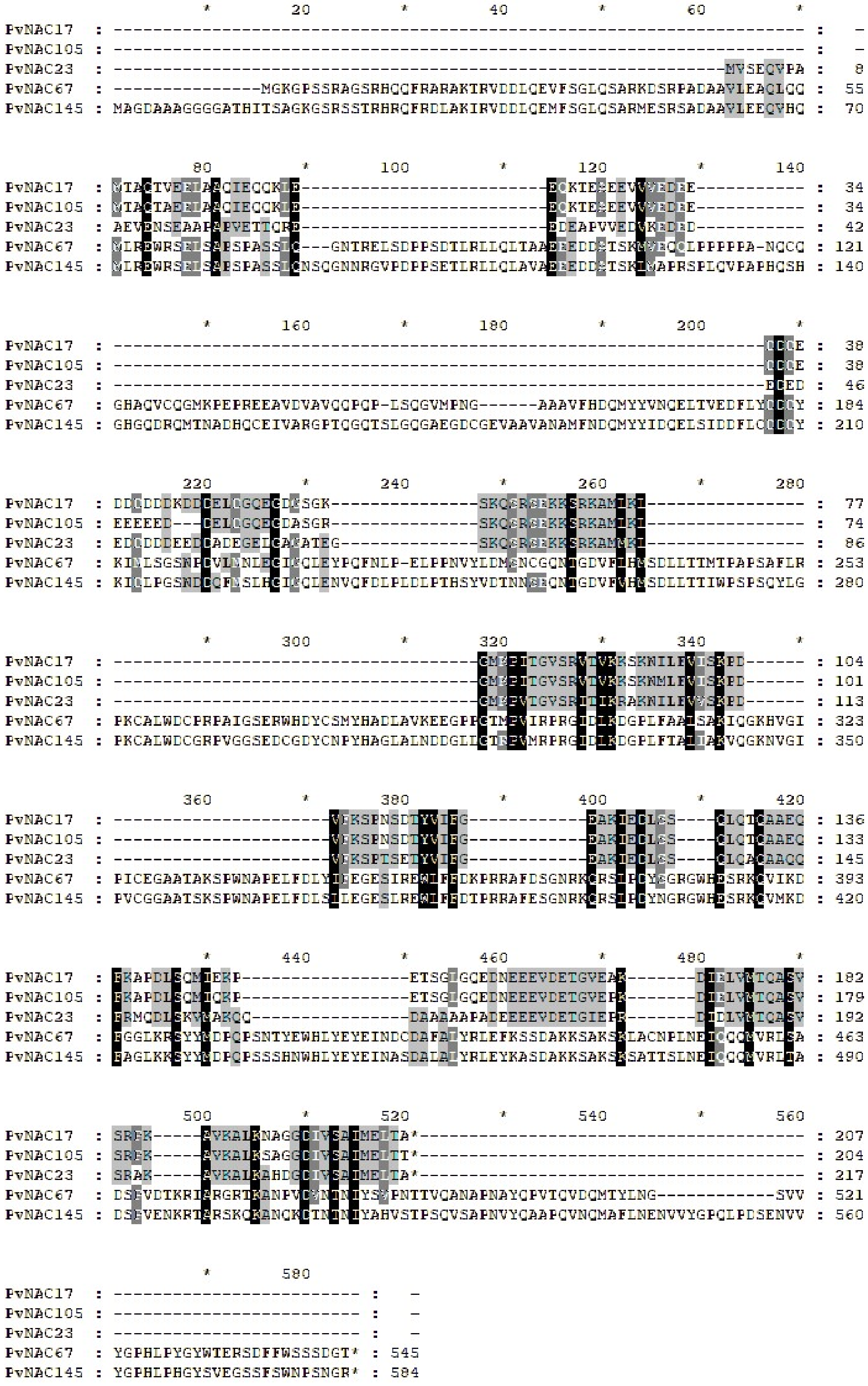

Supplement: Supplementary file 1 [file plants-13-03595-s001.zip › Figure S1.pdf]
