# Supplementary material for: Genome-Wide Identification and Expression Analysis of NAC Gene Family Members in Seashore Paspalum Under Salt Stress
Source: Plants (Basel). 2024 Dec 23;13(24):3595. doi: 10.3390/plants13243595 (PMC11678376; doi:10.3390/plants13243595)
Supplement: Supplementary file 1 [file plants-13-03595-s001.zip › Figure S2.pdf]

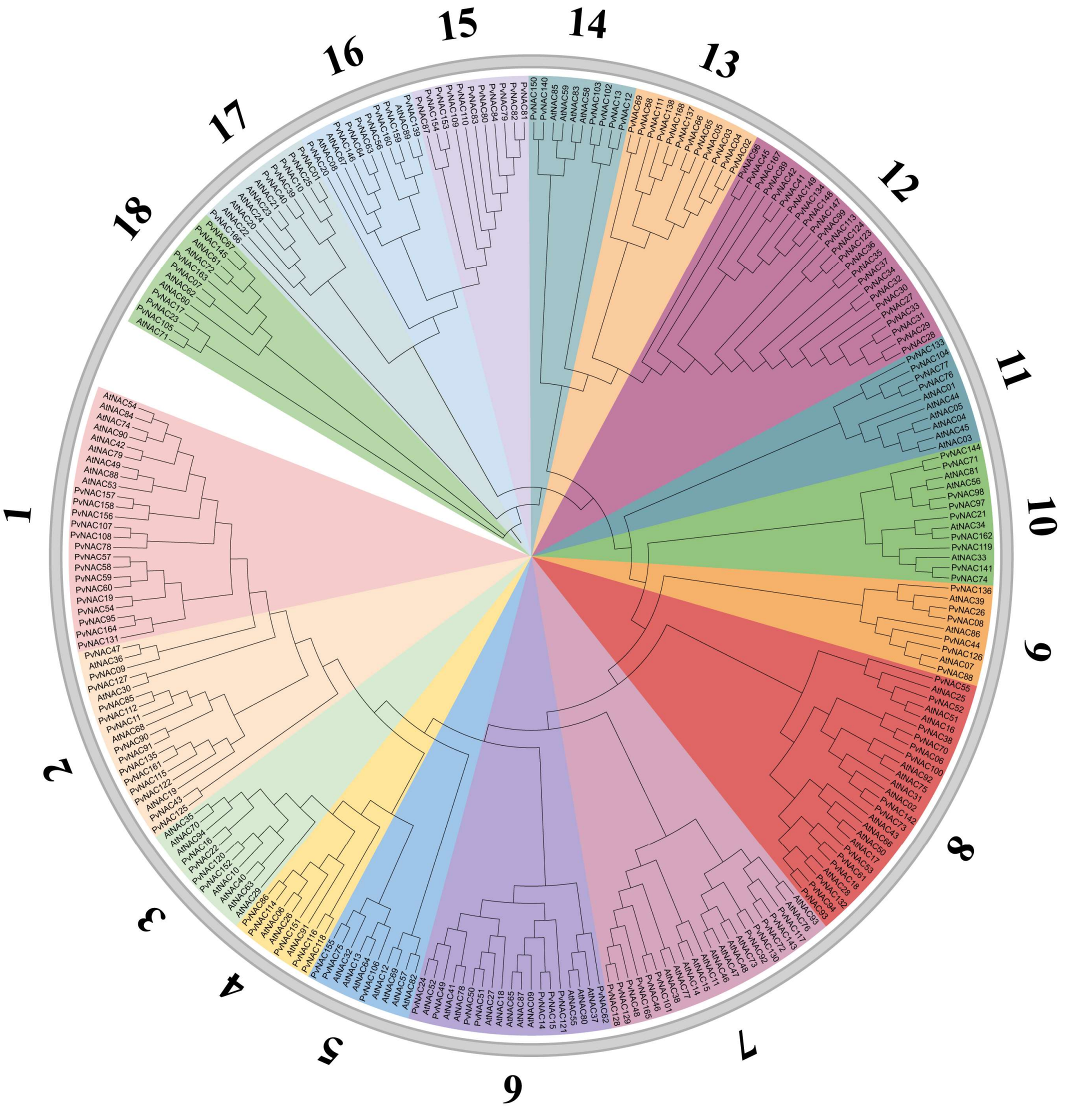

Supplementary Figure S2. Phylogenetic tree of NAC proteins derived from *Arabidopsis* and seashore paspalum. Distinct subgroups are highlighted in different colors, with clustering suggesting potential functional similarities among closely related proteins within each clade.
