# Supplementary material for: Genome-Wide Identification and Expression Analysis of NAC Gene Family Members in Seashore Paspalum Under Salt Stress
Source: Plants (Basel). 2024 Dec 23;13(24):3595. doi: 10.3390/plants13243595 (PMC11678376; doi:10.3390/plants13243595)
Supplement: Supplementary file 1 [file plants-13-03595-s001.zip › Figure S3.pdf]

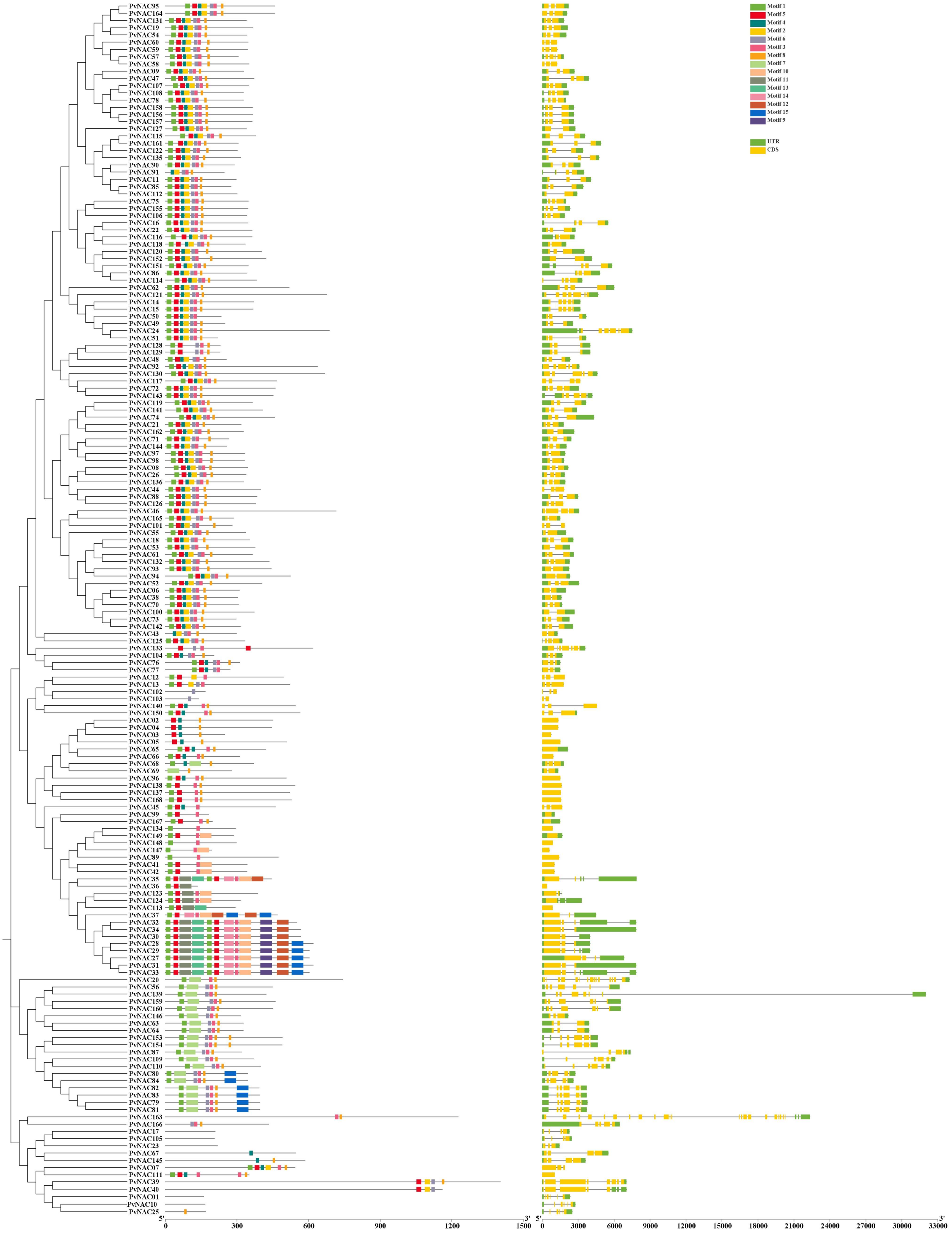

Supplementary Figure S3. Motif and *PvNAC* gene structure analysis. The left panel shows the motif analysis of *NAC* genes in seashore paspalum, with conserved motifs represented by colored boxes. The right panel illustrates the gene structure of *PvNAC*s, including exon-intron organization.
