# Supplementary material for: Genome-Wide Identification and Expression Analysis of NAC Gene Family Members in Seashore Paspalum Under Salt Stress
Source: Plants (Basel). 2024 Dec 23;13(24):3595. doi: 10.3390/plants13243595 (PMC11678376; doi:10.3390/plants13243595)
Supplement: Supplementary file 1 [file plants-13-03595-s001.zip › Figure S4.pdf]

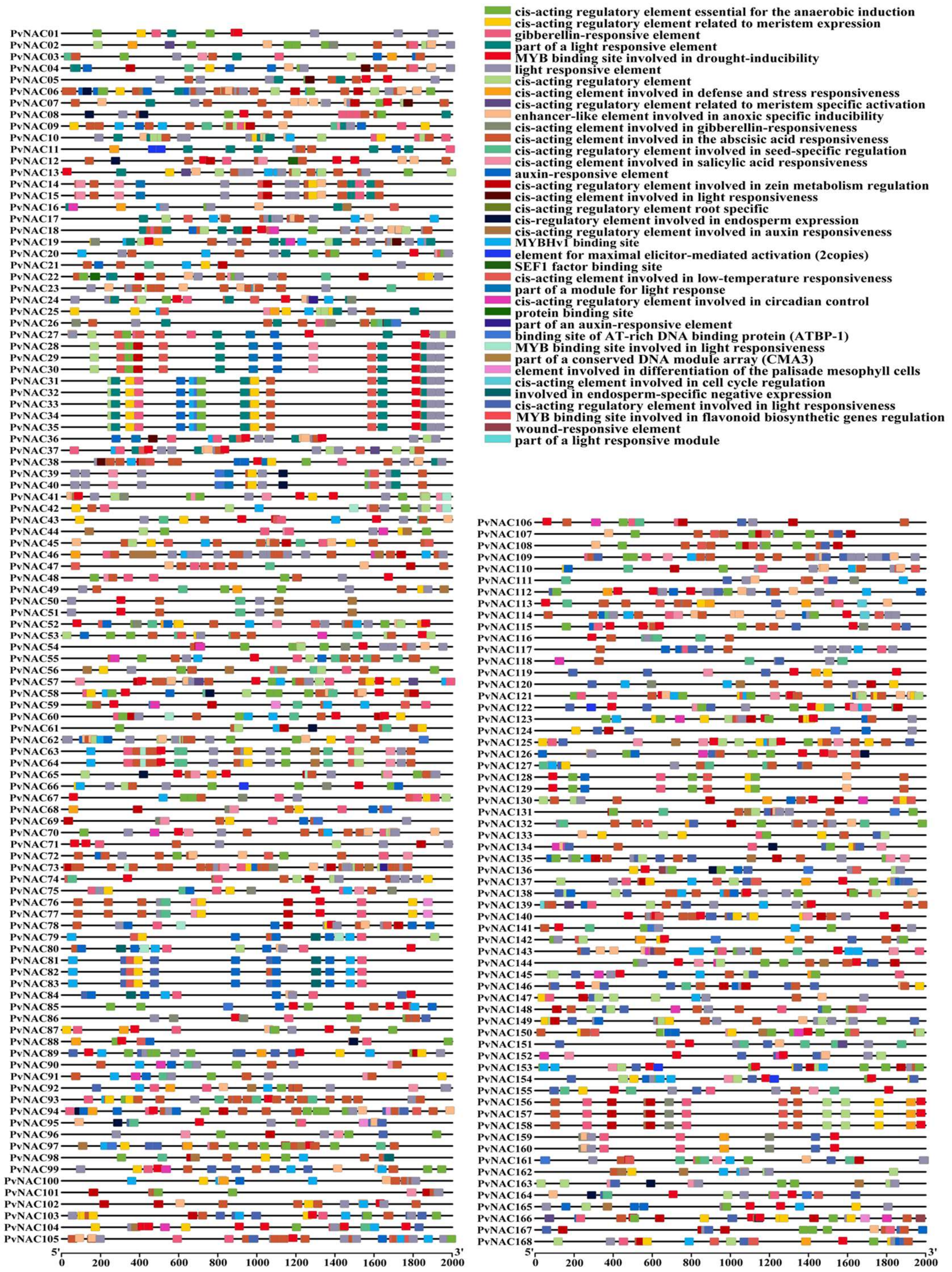

Supplementary Figure S4. Cis-element analysis of *PvNAC* transcription factors. This figure shows the distribution of cis-regulatory elements in the promoter regions of *PvNAC* transcription factors. Different colors represent various cis-elements associated with responses to environmental stress and hormones.
